# Socio-cultural factors influencing the prevention of mother-to-child transmission of HIV in Nigeria: a synthesis of the literature

**DOI:** 10.1186/1471-2458-14-771

**Published:** 2014-07-30

**Authors:** Juliet Iwelunmor, Echezona E Ezeanolue, Collins O Airhihenbuwa, Michael C Obiefune, Chinenye O Ezeanolue, Gbenga G Ogedegbe

**Affiliations:** Department of Kinesiology and Community Health, University of Illinois, Urbana-Champaign, 123 Huff Hall, 1206 S. Fourth St, Champaign, IL 61820 USA; Department of Pediatrics, University of Nevada School of Medicine, 2040 West Charleston Boulevard, Las Vegas, NV USA; Department of Biobehavioral Health, The Pennsylvania State University, 219 Biobehavioral Health Building, University Park, PA 16802 USA; Prevention, Education, Treatment, Training and Research-Global Solutions-PeTR-GS, 7 Link Road, Independence Layout Enugu, Enugu, 400001 Enugu State Nigeria; Sunrise Foundation, Plot 358 New GRA, Enugu, Nigeria; Center for Healthful Behavior Change, Division of General Internal Medicine, Department of Medicine, New York University School of Medicine, New York, USA

**Keywords:** Socio-cultural factors, PMTCT, PEN-3 cultural model

## Abstract

**Background:**

Currently, Nigeria alone accounts for 30% of the burden of mother-to-child transmission of HIV. This review explores the socio-cultural factors influencing prevention of mother-to-child transmission of HIV (PMTCT) service uptake in Nigeria.

**Methods:**

Using the PEN-3 cultural model as a guide, we searched electronic databases and conducted a synthesis of empirical studies conducted from 2001 to 2013 that reported the perceptions people have towards PMTCT, the enablers/resources that influence PMTCT service uptake, and the role of nurturers/family or community in shaping actions and decisions towards PMTCT service uptake.

**Results:**

A total of 42 articles meeting the search criteria were retained in this review. Thirty-six (36) were quantitative cross-sectional surveys; three were mixed methods, while three were qualitative studies. The findings highlight that there are perceptions, ranging from positive to negative that influence PMTCT service uptake in Nigeria. Furthermore, lack of available, accessible, acceptable, and affordable resources negatively influence decisions and actions towards PMTCT. Finally, family contexts matter with decisions and actions towards PMTCT service uptake in Nigeria particularly with disclosure and non-disclosure of sero-positive status, fertility intentions and infant feeding choices.

**Conclusion:**

As ambitious goals are established and unprecedented resources deployed towards the elimination of mother-to-child transmission of HIV globally by 2015, there is clearly a need to develop effective family-oriented, culture-centered community-based PMTCT programs in Nigeria so as to improve the low uptake of PMTCT services.

## Background

HIV infection among women of child-bearing age and mother-to-child transmission (MTCT) of HIV in Nigeria remain major health issues [1, 2]. The statistics are startling. In 2009, according to the Nigerian National Agency for the Control of AIDS (NACA), about 1.72 million women and girls were living with HIV and AIDS with the highest prevalence rate of 5.6% among women in the age group 25-29 [3]. In 2012, there were 110,000 new HIV infections among women aged 15-49 years in Nigeria, ranking the country second (next only to South Africa) among countries with the highest burden of new HIV infections among women [4]. Nigeria is one of 22 countries that account for 90% of pregnant women living with HIV [1]. In 2010, despite improved efforts dedicated to the prevention of mother-to-child HIV transmission (PMTCT), only 16.9% of pregnant women in Nigeria were tested for HIV [5]. The low rates of testing and treatment in Nigeria contributed to an estimated 75,000 HIV-infected infants in 2010 [1, 6]. In 2012, while these rates dropped, 60,000 new HIV infections occurred among children, now making Nigeria the country with the largest number of children acquiring HIV through MTCT [4]. Currently, Nigeria alone, "*accounts for 30% of the burden of MTCT"* [7] *.* Without urgent action in Nigeria, UNAIDS suggests that the global target of eliminating new HIV infections among children by 2015 is unlikely to be reached [4].

The World Health Organization (WHO) along with key strategic partners is fully committed to the prevention-of-mother-to child transmission of HIV through; primary prevention of HIV infection among women of child-bearing age, preventing unintended pregnancies among women living with HIV and scaling up access to effective PMTCT services that prevent HIV transmission from a woman living with HIV to her infant, and provides appropriate treatment, care and support for mothers living with HIV, their children and their families [8]. In addition, effective PMTCT interventions, particularly in resource-poor settings, have the potential to avert pediatric infections and save numerous lives. As a result, current national targets in Nigeria include ensuring that at least 80% of all pregnant women have access to voluntary counseling and testing, 80% of all HIV-exposed infants have access to early infant diagnosis services, 80% of all HIV-positive pregnant women and HIV-exposed infants have access to efficacious ARV’s and 80% of HIV-positive pregnant women have access to infant feeding counseling by 2015 [9]. If these targets are to be met by 2015, identifying the socio-cultural factors that influence pregnancy, labor, delivery or breast-feeing are important for the success of PMTCT efforts in Nigeria. Moreover, since culture plays a vital role in determining the level of health of the individual, family, and community [10], it is plausible that knowledge of socio-cultural factors may facilitate efforts aimed at effective PMTCT in Nigeria. This in turn, may reduce missed opportunities including late initiation of prenatal care, low perceptions of risk, failure to disclose knowledge of HIV sero-positive status, and lack of knowledge of the effectiveness of interventions for PMTCT.

Using a cultural model as a guide, this paper reviews the current information available on the socio-cultural factors that influence PMTCT in Nigeria. The implications are discussed in light of ambitious goals and unprecedented resources currently in place to virtually eliminate mother-to-child transmission of HIV by 2015. Interventions focused on addressing the influence of socio-cultural factors with PMTCT efforts in Nigeria are also discussed.

### Theoretical framework: the PEN-3 cultural model

**Figure 1:**
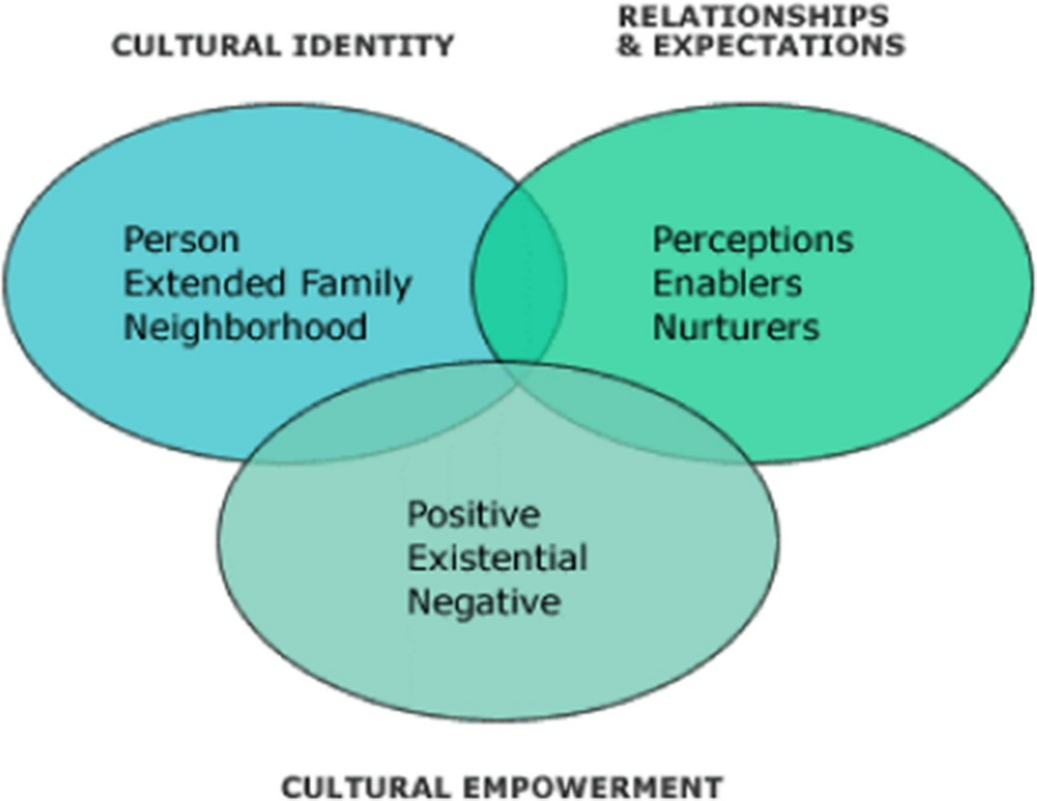
**The PEN-3 cultural model.**

Developed by Airhihenbuwa, the PEN-3 cultural model addresses the role of culture in the adoption or non-adoption of health behaviors [10, 11]. It consists of three domains (see Figure 1); Cultural Identity, Relationships and Expectations, and Cultural Empowerment, that offer the opportunity to centralize culture in the development of health promotion interventions [10]. Each domain includes three factors that form the acronym PEN; **P**erson, **E**xtended Family, **N**eighborhood (Cultural Identity domain); **P**erceptions, **E**nablers, and **N**urturers (Relationship and Expectation domain); **P**ositive, **E**xistential and **N**egative (Cultural Empowerment domain).

The PEN-3 cultural model also serves as a tool for examining the context that shapes the health behavior of interest. This is achieved by highlighting culturally relevant factors that are influential and should be considered in the development of effective targeted health interventions [12]. Since its inception in 1989, the PEN-3 cultural model has been used to develop effective outreach cancer control interventions for Latinos [12, 13], diabetes prevention interventions for African Americans [14], culturally competent domestic violence interventions and preventive services for Chinese immigrants [15], and to conduct community based research on HIV Stigma in South Africa [16]. In this paper, we will focus on the Relationship and Expectations domain as well as the Cultural Empowerment domain of the PEN-3 model. With the Relationships and Expectations domain, findings from the literature will be categorized into Perceptions or attitudes about PMTCT, Enablers or societal/structural resources such as health care services that promote or discourage effective health seeking practices for PMTCT, and Nurturers such as the influence of family and kin in nurturing decisions surrounding effective participation in PMTCT programs are examined. Findings from the literature will also be categorized based on the Cultural Empowerment domain as either Positive, Existential (unique) or Negative. With the Cultural Empowerment domain, Positive refers to key values that promote PMTCT service uptake; Existential are unique values practiced in the culture that pose no threat to PMTCT service uptake; while Negative includes health beliefs and actions that are harmful and negatively influence decisions or actions to obtain PMTCT services or create barriers to service uptake. Overall, using the PEN-3 cultural model as a guide provides the opportunity to identify socio-cultural perceptions, enablers, or nurturers that are positive, acknowledges the ones that are existential, before identifying the negative. In this way, effective culture-centered intervention for PMTCT is as much about promoting these positive values as it is changing negatives ones.

## Methods

**Figure 2:**
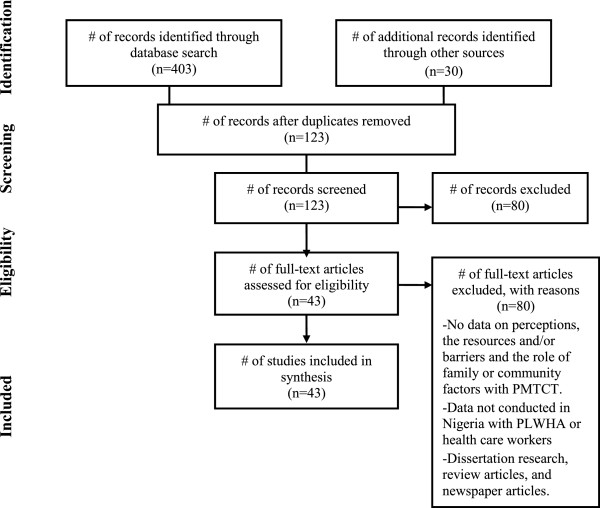
**PRISMA flow diagram of literature reviewed.**

We reviewed all published abstracts and journal articles from 2001 to 2013 conducted in Nigeria. The year 2001 was chosen as it was the year the Federal Government of Nigeria adopted the UNGASS (United Nations General Assembly Special Session) goal of reducing the proportion of infants infected with HIV by 20% by 2005 and 50% by 2010," as well as increasing universal access to quality voluntary counseling and testing by 50% by 2010. A comprehensive search of 5 electronic database; Embase, Google Scholar, JSTOR, PubMed and Sociologic Abstracts were utilized in this review. The review methodology was also adapted from other reviews on PMTCT in sub-Saharan Africa so as to ensure the strength of the assessment [17]. The search terms used for this review contained keywords or texts within a matrix of relevant terminology (e.g. PMTCT + Nigeria, HIV testing + Pregnant Women + Nigeria etc.). Reference list of relevant articles were also searched to identify available literature. The abstracts of all of the documents were initially screened by one reviewer; however, the full-text documents were retrieved and re-screened by two reviewers independently. To be eligible for this review, the article must have included data on socio-cultural factors influencing PMTCT. Specifically, the article must have identified perceptions people may have towards PMTCT, the resources and/or barriers that influence PMTCT service uptake and the role of family or community factors in shaping decisions surrounding participation in PMTCT programs. The study must have also been conducted in Nigeria. The synthesis of the data extracted from the available literature was also conducted using the PEN-3 cultural model as a guide. Also, the quality of included papers were assessed using the standard checklist for assessing the quality of research papers [18]. Specifically, we assessed whether the objectives of the paper where sufficiently described, whether study design was evident and appropriate, whether information on target population was provided, whether data collection methods and analysis were clearly described, whether results were reported in a sufficient manner and whether the conclusion were supported by the results [18]. The searches yielded studies that varied greatly in terms of their sample size, study design, and objectives (see Table 1). Also, PRISMA flow diagram of the literature reviewed is presented in Figure 2.

**Table 1:** **Summary of Literature Reviewed**

Reference	Setting (Urban/Rural)	Sample	Study Type	Aim
Nwakwuo et al., 2013 [19]	Semi-Urban	400 households	Cross-sectional survey	To assess the level of male involvement in their spouses' reproductive health events before pregnancy, during pregnancy, delivery and peuperium
Ogbolu et al., 2013 [20]	Rural/Urban	231 nurses	Cross-sectional survey	To examine current PMTCT practices in 27 public health facilities in Nigeria.
Olugbenga-Bello et al., 2013 [21]	Urban	420 women (15-49 years)	Cross-sectional survey	To assess knowledge and attitude of women of child-bearing age towards PMTCT
Amoran et al., 2012 [22]	Urban	225 pregnant women	Cross-sectional survey	To assess factors associated with the knowledge and utilization of PMTCT services by the teenage pregnant women when compared to mature pregnant women.
Hembah-Hilekaan et al., 2012 [23]	Semi-Urban	384 women	Cross-sectional survey	To assess knowledge, attitudes and barriers to the uptake of PMTCT
Nwabueze et al., 2012 [24]	Semi-Urban	288 women	Cross-sectional survey	To assess the determinants of subjective health status of HIV-positive mothers assessing PMTCT services
Ogbe et al., 2012 [25]	Urban	140 women	Cross-sectional survey	To determine the contraceptive awareness among HIV positive women
Sofolahan et al., 2012 [26]	Urban	60 women	In-depth interviews	To understand the factors responsible for the childbearing decisions of women living with HIV/AIDS
Ugwu et al., 2012 [27]	Urban	150 antenatal clients	Cross-sectional survey	To study the impact of health education on the awareness of strategies for PMTCT
Bello et al., 2011 [28]	Urban	104 women	Cross-sectional survey	To assess the acceptability and suitability of offering HIV counselling and testing to women of unknown HIV status presenting in labour.
Ezegwui et al., 2011 [29]	Urban	96 HIV positive pregnant women	Cross-sectional survey	To evaluate sexual behavior and activity in HIV positive pregnant women and their sources of information
Ezeanochie et al., 2011 [30]	Urban	305 HIV positive women	Cross-sectional survey	To evaluate the prevalence and correlates of intimate partner violence among HIV-positive pregnant Nigerian women.
Olagbuji et al., 2011 [31]	Urban	166 HIV positive women	Cross-sectional survey	To determine the prevalence, pattern and determinants of spousal disclosure of HIV serostatus
Balogun et al., 2010 [32]	Urban	108 Traditional Birth Attendants	Cross-sectional survey	To assess the knowledge and practice of PMTCT amongst TBAs in Lagos, Nigeria.
Enwereji et al., 2010 [33]	Semi-Urban	96 PLWHA and 45 healthcare workers	Mixed-method	To identify factors and conditions that determine childbirth choices of PLWHA
Oladokun et al., 2010 [34]	Urban	51952 women at antenatal clinic	Cross-sectional survey	To evaluate the service uptake and performance of PMTCT program using national key indicators
Oladokun et al., 2010 [35]	Urban	241 women	Cross-sectional survey	To evaluate the infant-feeding choices, practices and possible determinants among HIV-positive women enrolled in a PMTCT program
Adeleke et al., 2009 [36]	Urban	164 mothers	Cross-sectional survey	To evaluate the awareness and knowledge of mother-to-child transmission of HIV, HIV/AIDS and the methods to prevent mother-to-child transmission of HIV.
Brown et al., 2009 [37]	Urban	513 mothers	Cross-sectional survey	To evaluate breastfeeding and weaning practices associated socio-demographic factors and knowledge about mother-to-child transmission of HIV among mothers
Ezechi et al., 2009 [38]	Urban	652 HIV positive pregnant women	Cross-sectional survey	To determine the prevalence, types and correlates of intimate partner violence (IPV) in pregnant Nigerian living with HIV.
Ezegwui et al., 2009 [39]	Urban	92 pregnant women	Cross-sectional survey	To assess HIV serostatus disclosure pattern among pregnant women
Maru et al., 2009 [40]	Urban	469 women	Mixed-methods	To identify the social determinants of mixed feeding
Moses et al., 2009 [41]	Urban	172 women	Cross-sectional survey	To determine the level of knowledge, practice and attitude toward HIV/AIDS issues with respect to PMTCT
Mukhtar-Yola et al., 2009 [42]	Urban	190 HIV exposed babies	Cross-sectional survey	To determine the sociodemographic characteristics, infant feeding choices and outcome of HIV exposed neonates
Omuemu et al., 2008 [43]	Urban	200 pregnant women	Cross-sectional survey	To assess the awareness, attitude and practice of HIV testing among antenatal clients
Onah et al., 2008 [44]	Urban	635 pregnant women	Cross-sectional survey	To assess voluntary counselling and testing (VCT) uptake, nevirapine use and infant feeding options among pregnant women
Sadoh et al., 2008 [45]	Urban	103 mothers	Cross-sectional survey	To evaluate the feeding practices of HIV-infected mothers in the first six months of their infants’ lives
Arulogun et al., 2007 [46]	Urban	20 community gatekeepers	In-depth interviews	To identify level of awareness and knowledge of PMTCT services
Okonkwo et al., 2007 [47]	Semi-Urban	240 pregnant women	Cross-Sectional Survey	To determine the awareness, attitudes, and beliefs of pregnant Nigerian women toward voluntary counseling and testing (VCT) for HIV
Adeneye et al., 2006 [48]	Urban	804 women at antenatal clinic	Mixed methods	To assess willingness to seek and undergo HIV counseling and testing.
Daniel et al., 2006 [49]	Urban	333 pregnant women	Cross-sectional survey	To assess the acceptability of prenatal HIV screening
Ekabua et al., 2006 [50]	Urban	400 women at antenatal clinic	Cross-sectional survey	To determine the level of awareness, attitude and practice of antenatal HIV screening
Sagay et al., 2006 [51]	Urban	570 HIV positive mothers	Cross-sectional survey	To explore the issues concerning disclosure of HIV status to partners of HIV sero-positive mothers in a PMTCT programme
Sagay et al., 2006 [52]	Urban	500 partners of HIV infected pregnant women	Cross-sectional survey	To determine the pattern of HIV sero-status of Partners of HIV Positive Pregnant Women
Igwegbe et al., 2005 [53]	Semi-Urban	312 pregnant women at antenatal clinic	Cross-sectional survey	To evaluate the knowledge and perceptions of HIV/AIDS and mother to child transmission among pregnant women
Iliyasu et al., 2005 [54]	Urban	210 women at antenatal clinic	Cross-sectional survey	To assess pregnant women's knowledge of HIV/AIDS, awareness and attitudes towards Voluntary Counselling and Testing (VCT) in a teaching hospital in northern Nigeria.
Oladapo et al., 2005 [55]	Semi-Urban	147 HIV positive men and women	Cross-sectional survey	To determine the extent of fertility desires and intentions of HIV-positive patients
Chama et al., 2004 [56]	Urban	262 pregnant women at antenatal clinic	Cross-sectional survey	To determine uptake of PMTCT services at an antenatal clinic
Ekanem et al., 2004 [57]	Urban	345 pregnant women at antenatal clinic	Cross-sectional survey	To determine their knowledge and acceptability of HIV voluntary counselling and testing in pregnancy
Fasubaa et al., 2001 [58]	Semi-Urban	586 pregnant women at antenatal clinic	Cross-sectional Survey	To assess pregnant clients' opinions on the issue of antenatal HIV screening
Orji et al., 2001 [59]	Semi-Urban	200 pregnant women at antenatal clinic	Cross-sectional survey	To determine the attitude of pregnant women to routine HIV screening
Owolabi et al., 2001 [60]	Semi-Urban	4 HIV positive pregnant women	Case-Study	To highlight the socio-economic implications and the burden of HIV on maternal and child health

## Results

Forty-two quantitative, mixed-methods and qualitative papers published between 2001 and 2013 from urban, semi-urban, and rural areas of Nigeria which met our inclusion criteria were included in the synthesis.

Thirty-six (36) were quantitative cross-sectional surveys; three were mixed methods, while three were qualitative studies. Majority involved women, particularly HIV positive women attending ante-natal clinics throughout Nigeria. The initial screening characterized the data into the Relationship and Expectations domain and/or the Cultural Empowerment Domain. The emerging themes were then re-organized by cross-tabulating the Relationship/Expectations domain with the Cultural Empowerment domain (see Table 2). This approach, known as the assessment phase of the PEN-3 model, provide the opportunity to arrange the emerging themes into perceptions, enabling factors and nurturing factors influencing PMTCT in Nigeria.

**Table 2:** **Themes identified using the PEN-3 cultural model**

	Themes from the literature reviewed	Positive	Existential	Negative
Perceptions	Knowledge of HIV transmission	X		
	Acceptance of VCT	X		
	Willingness to go for VCT	X		
	Desire to have children		X	
	Desire to breastfeed		X	
	Poor condom use knowledge			X
	Poor awareness of ARV’s			X
	Poor awareness of PMTCT			X
	Stigma and discrimination			X
	HIV = death, abandonment, rejection			X
Enablers	Confidentiality of HIV test results	X		
	PMTCT Health education programs	X		
	Family planning services, HAART	X		
	Support group and counseling	X		
	Support group and antiretroviral therapy	X		
	Traditional Birth Attendant		X	
	Inadequate VCT and PMTCT centers			
	Long distance, long waiting times			X
	Financial difficulties and cost			X
	Stigma			X
	Discrimination from health workers			X
	Lack of confidentiality of results			X
	Refusal of HIV testing			X
Nurturers	Disclosure of status and support	X		
	Male support and involvement	X		
	Family support and infant feeding choice	X		
	Family support and ARV adherence	X		
	Ensure lineage continuity and posterity		X	
	Non-disclosure of seropositive status			X
	Desire not to procreate			X
	Stigma			X
	Family pressure to breastfeed			X

### Perceptions towards PMTCT in Nigeria

In this paper, perceptions include positive, existential and/or negative knowledge, attitudes, and beliefs that influence decisions and actions towards PMTCT in Nigeria. Table 2 highlights the perceptions identified from the studies reviewed. There was a high level of acceptance with HIV testing, with some participants suggesting ‘*that pregnant woman should be tested for HIV’* [21, 22] coupled with a *‘willingness to go for voluntary counseling and testing (VCT)’* [48, 57]. These positive perceptions appeared to be influenced by an increased awareness that *‘VCT could reduce the risk of transmission of HIV to babies’* [47] *,* as well as by *‘increasing educational level, not fearing a blood test; perception that clinics offered privacy; and perceptions of higher levels of social support from relatives and peers’* [48] *.* Unique or existential perceptions reflect values or beliefs that help to explain practices in the Nigerian culture and could influence attitudes towards PMTCT. In the literature reviewed, *‘the desire to have children’* was a common existential perception shared by many HIV positive men and women in Nigeria [26, 33, 61]. Culture, particularly the strong cultural attachment to having children, appeared to influence this desire for childbearing among HIV positive men and women. For example, in a study on the reproductive intent of HIV positive men and women in southeastern Nigeria, the authors found that ‘*broadly held social expectations with regard to reproduction are experienced even more acutely by HIV-positive people’* [62]. Another study found that women with non-formal education were more likely to desire having children than women with tertiary education [61]. Similarly, the desire to breastfeed was a common existential perception shared by HIV positive women in some studies. One study found that irrespective of HIV status, some women reported that they would still breastfeed their children [57]. Nevertheless, in the literature reviewed, lack of knowledge on condom use [22], the purpose of ARV despite awareness of it [33, 57] as well as poor knowledge with regards to PMTCT [46, 48, 57] were among the major negative perceptions identified. For example, in a study conducted among pregnant women in Western Nigeria, the authors found that participants *‘did not know how to correctly use condom to prevent pregnancy and HIV/AIDS infection’* [22]. In southeastern Nigeria, one study found a significant number of HIV positive women who discontinued use of iron tablets and/or ARV drugs because they feared it may lead to ‘*big* babies’ and ‘*threatened abortion’* [33]. Among participants in southwestern Nigeria, some believed that termination of pregnancy could be a method of preventing mother-to-child transmission of HIV [21]. In determining patterns and determinants of ARV’s during pregnancy, researchers [63] found that perceptions of stigma and discrimination such as the *‘fear of being identified as HIV positive’* was the most common reason given for non-adherence. Perceptions of stigma and discrimination were frequently observed in majority of the studies reviewed with researchers suggesting these are also deterrents to VCT for some pregnant women [33, 47, 63]. Other negative perceptions identified in the literature were the synonymous linking of HIV with death, abandonment and rejection [33].

### Enabling factors influencing PMTCT in Nigeria

In the literature reviewed, enablers refer to the availability, accessibility, acceptability, and affordability of resources that either facilitate or hinder decisions and actions towards PMTCT. For example, confidentiality of HIV test results, availability of health education programs surrounding PMTCT and access to family planning services, HAART, support groups, and counseling services for HIV positive women were some of the positive enablers identified in the literature [64]. In an evaluation of pregnant women’s attitudes towards VCT in Awka, Nigeria, the authors found that assurance of confidentiality of results influenced willingness to test for HIV [47]. Also, in Enugu, Nigeria, researchers found a positive and significant impact of health education on the awareness and acceptability of strategies for PMTCT [27]. Another study suggested that establishment of a community peer support group for all nursing mothers (to prevent stigmatization) may assist in changing community norms and beliefs while providing support for HIV positive mothers, especially young mothers who choose to exclusively breastfeed their children [65]. While, early initiation of PMTCT interventions, including the access to more [‘counseling sessions during the beginning of pregnancy provided enabling environments for mothers to carry out their infant feeding intentions [65].

Some enablers identified in the literature are also existential, in that they are traditionally available in the community or society and support uptake of PMTCT services. For example, some women living with HIV and AIDS utilized the services of traditional birth attendants before, during, and after pregnancy [32]. Throughout Nigeria, irrespective of HIV status, about 60% of childbirths are performed by traditional birth attendants [20, 32]. Among HIV positive pregnant women, some of the reasons given for assessing the services of traditional birth attendants include the unfriendly attitudes of healthcare workers and the cost of childbirth. Also, since the experience of childbirth is governed by numerous traditional birthing practices, traditional birth attendants were preferred because they provide important information on the local customs, traditions, and perceptions regarding childbirth and new-born care. Balogun and Odeyemi suggest that *‘both rural and urban women seek care with traditional birth attendants because they have similar cultural and socio-economic characteristics’* [32].

Nevertheless, lack of available, accessible, acceptable, and affordable resources negatively influence decisions and actions towards PMTCT. For example, across the studies reviewed, inadequate and inaccessible voluntary and counseling centers, coupled with long distance to the centers and long waiting lines acted as barriers to PMTCT service uptake. In a study conducted in the Aminu Kano Teaching Hospital in Kano in which the records of HIV positive infants were reviewed, the authors found that inaccessibility to PMTCT centers were among the reasons their mother gave for non-participation in PMTCT program [66]. Further financial difficulties and cost, particularly costs associated with childbirth influenced decisions to participate in PMTCT services at health clinics. This was the case in southeastern Nigeria where researchers observed that high treatment costs influenced the childbirth choices of HIV positive pregnant women [33]. Also, stigma and discrimination from nurses, including non-acceptance, unfriendly attitudes and lack of confidentiality were deterrents in the non-utilization of hospital services [33, 66].

### Nurturing factors influencing PMTCT in Nigeria

In the literature reviewed, nurturers refer to the influence of family and/or community contexts in positively or negatively shaping decisions and actions towards PMTCT service uptake in Nigeria. Indeed, disclosure of sero-positive status and subsequent support from family members defined some of the positive nurturers’ category. These findings are consistent with studies conducted elsewhere which suggests that family and community systems remain the first and best line of support for caring and supporting family members who are living with HIV and AIDS [67]. In Northern Nigeria, one study observed supportive reactions to HIV sero-positive status among partners of HIV positive women [52]. This was also the case in Enugu, where another study observed a high level of sero-status disclosure of HIV status, with most women disclosing to their partners, and receiving support [39]. Family support which is important in PMTCT programmes also provided optimal access to preventive strategies such as adherence to ARV therapy. For example, in Lagos, disclosure of HIV status and subsequent treatment support from partners was reported to influence the level of adherence to ARVs among pregnant women [63]. Disclosure of sero-positive status to partners also influenced the choice and maintenance of infant feeding [35, 40].

In addition to these positive nurturing factors, the literature reviewed revealed certain traditional values and practices influenced by family contexts that shape PMTCT decisions or health-seeking behaviors. For example, with the widespread use of ARV’s for the treatment of HIV, as indicated earlier, ‘the desire to have children’ has become a common existential perception shared by many HIV positive men and women living in Nigeria. Participants in some studies reported that the main reasons for wanting to procreate included ensuring lineage continuity and posterity [26, 33, 61]. These desires seemed to be influenced by a strong cultural attachment placed on having children, coupled with the stigma associated with childlessness, role of children in inheritance, importance of children in agricultural economies, importance of childbearing on the status of women and the role of children as caretakers of the elderly [61].

Nevertheless, there were factors influenced by family contexts that negatively shaped decisions and actions towards PMTCT. For example, while some participants willingly disclosed knowledge of their sero-positive status to their partners, in some studies, researchers also observed non-disclosure of sero-positive status. In southeastern Nigeria, one study observed that the fear of divorce, alongside being single, low educational status, Anglican Christian denomination and non-membership in a support group significantly increased the likelihood of non-disclosure of sero-positive status [68]. Similarly, among HIV positive mothers in Benin City Nigeria, three common reasons given for non-disclosure was *‘fear, regarding the spread of the information, stigma and deterioration in the relationship with the spouse’* [31]. Non-disclosure of sero-positive status also influenced non adherence to ARV’s among HIV-positive pregnant women in Nnewi Nigeria [69]. Among women who disclosed their HIV status, partner testing was found to be dismal in many cases. In an evaluation of PMTCT service uptake among 2152 HIV positive women attending a PMTCT programme in Ibadan, Nigeria, only about 16% of their partners accepted HIV testing [34]. Also, while there was evidence for a strong desire to procreate given the strong cultural attachment to having children in Nigeria, findings from the literature reviewed also indicated that knowledge of sero-positive status in some cases, influenced decisions not to reproduce. For example, in a study conducted among HIV positive men and women in the Niger Delta Region, the authors found that decisions not to have more children were linked to the fear of infecting a sero-discordant partner and baby, fear of dying and leaving behind orphans, and fear that they may become too ill and unable to support the child financially [61]. For new HIV positive mothers, stigma and family pressure to breastfeed influenced mixed feeding patterns. One study observed that family members input with regards to culturally and socially accepted feeding methods, alongside poor access to proper feeding counseling support influenced high rate of mixed feeding practices in a cohort of HIV positive mothers in the south-south region of Nigeria [70].

## Discussion

There are nearly 1.72 million women and girls living with HIV in Nigeria and the prevalence continues to increase despite efforts by the Nigerian Ministry of Health [3]. Furthermore, 60,000 new HIV infections occurred among children, making Nigeria the country with the largest number of children acquiring HIV through mother-to-child transmission of HIV (MTCT) [7]. If the national targets of ensuring that at least 90% of all pregnant women have access to voluntary counseling and testing, 80% of all HIV-exposed infants have access to early infant diagnosis services, 80% of all HIV-positive pregnant women and HIV-exposed infants have access to efficacious ARV’s and 80% of HIV-positive pregnant women have access to infant feeding counseling are to be met by 2015 [9], then it is essential to identify the socio-cultural factors that facilitate or hinder PMTCT service uptake in Nigeria.

The findings from the literature reviewed demonstrate that socio-cultural factors matter with PMTCT service uptake in Nigeria. Three main conclusions can be drawn from the literature reviewed. First, there are perceptions, ranging from positive to negative that influence PMTCT service uptake in Nigeria. While we observed a high-level of acceptance for HIV testing and willingness to undertake voluntary counseling and testing, there continues to be negative perceptions, such as the fear of stigma and discrimination that influenced decisions and actions towards PMTCT service uptake such as HIV testing [47]. This finding is consistent with scholarly assertions which suggest that stigma and discrimination are major deterrents to voluntary counseling and testing for HIV [71]. For example, studies conducted in other parts of Africa highlighted the negative effects of HIV stigma and discrimination and suggested that it impedes efforts to promote voluntary counseling and testing as well as the provision of treatment, care and support [16, 71]. In Nigeria, perceptions of stigma and the fear regarding spread of sero-positive status also influenced non-disclosure of HIV sero-positive status [31] and non-adherence to ARV’s among HIV positive pregnant women [33]. If mother-to-child-transmission of HIV is to be effectively reduced in Nigeria, there is a continued need to examine the broader socio-cultural contexts in which stigma and discrimination occurs so as to develop effective culture-centered interventions focused on reducing stigma in PMTCT programs. Furthermore, routinely offering HIV tests to all pregnant women and their partners and integrating HIV testing with other prenatal tests and into other services will help further reduce the stigma and discrimination associated with HIV-only test approach.

A second, main conclusion that can be drawn from the literature is the role of enablers (resources) relative to societal or systemic factors in facilitating or hindering efforts to participate effectively in PMTCT programs. Positive enablers such as assurance of confidentiality results influenced willingness to test for HIV. Furthermore, health education programs were linked to acceptability of PMTCT programs, and participation in community support programs significantly influenced infant feeding choices. Negative enablers such as inadequate and inaccessible VCT, structural constraints at health centers including long distance to clinic and long waiting lines, coupled with financial difficulties and perceptions of stigma and discrimination greatly reduced uptake of PMTCT programs in Nigeria. The success of PMTCT efforts in Nigeria, particularly with reducing missed opportunities will rely on identifying culturally compelling ways to promote the positive enabling factors while changing the negative ones. Furthermore, findings from the literature indicated that efforts should be made to effectively integrate traditional birth attendants with prenatal, child delivery and postnatal care in Nigeria. Indeed, studies conducted elsewhere suggested that traditional birth attendants are critical with reducing missed opportunities for PMTCT particularly as their services are utilized by pregnant women not currently receiving formal antenatal care and they provide assistance during delivery and postpartum care [72, 73]. Since up to 60% of pregnant mothers rely on the services of traditional birth attendants [20, 32], recognizing their cultural and practical contribution is crucial for the success of PMTCT efforts in Nigeria.

The third conclusion drawn from the literature reviewed suggests that family contexts matter with decisions and actions towards PMTCT in Nigeria. Indeed family systems demonstrated remarkable capacities to adjust to the burden of HIV by readily providing support for family members living with HIV particularly following disclosure of sero-positive status [67]. The support provided by family members also influenced adherence to ARV’s among pregnant women and infant feeding choice. In addition, the desire to procreate, an existential perception shared by many of HIV positive men and women in the literature reviewed was influenced by the strong cultural attachment placed on having children particularly with ensuring lineage continuity and posterity. Nonetheless, while there were positive and existential nurturing factors that influenced decisions and actions towards PMTCT service uptake, we also identified negative factors that hinder service uptake. These factors such as non-disclosure of sero-positive status to significant others were influenced by perceptions of fear, stigma and discrimination. Failure to disclose also influenced non-adherence to ARV’s and hindered PMTCT completion. Nevertheless, in cases where HIV positive women disclosed, partner testing for HIV was low. This is similar to findings reported in other parts of sub-Saharan Africa [74]. As a result, better culturally appropriate strategies are needed to effectively involve men in PMTCT efforts in Nigeria.

One strategy is to develop family-oriented, sustainable community-based programs that offer PMTCT services including voluntary counseling and testing services outside clinic settings. For example, congregation-based PMTCT programs such as the Healthy Beginning Initiative (HBI) in Southeast Nigeria [1] could serve as a model in that it utilizes the church network to decentralize testing beyond health facilities while improving access to testing and treatment services. A congregation-based approach to PMTCT in Nigeria is ideal for several reasons. First, faith plays an important role in the life of Nigerians. Ranked #1 among 53 other nations in church attendance at 89%, Nigeria has extensive penetration with faith-based institutions and faith plays a significant role in the social life of Nigerians [1, 75]. The ubiquity of religious institutions in Nigeria and the growing involvement of faith-based organizations in HIV prevention present a unique opportunity to employ, and evaluate the effect of congregation-based approaches on HIV testing among pregnant women, male partners of pregnant women and PMTCT completion rate among HIV-infected pregnant women [1]. A congregation-based approach holds great potential and has been used effectively in health promotion and disease prevention in communities where religion plays a prominent role [76–79]. This strategy may also be useful with reducing missed opportunities for PMTCT including perceptions of stigma and/or family pressure to engage in infant feeding methods. Moreover, this approach could easily be scaled up in any congregation setting and could serve as a model for other interventions aimed at improving maternal-child health outcomes [1].

## Conclusion

### Limitations

There are several limitations worth noting. First the generalizability of our findings is limited to PMTCT efforts within the Nigerian context. Second, our findings should be interpreted cautiously in the light of the sources of the data, variability in the methodologies used to collect data and even the use of the PEN-3 cultural model. Indeed, because most of the studies reviewed employed a cross-sectional design, causality among variables cannot be assumed. Also, since we limited our synthesis of the literature to only peer-reviewed literature in electronic databases, reliance on these sources prevents us from reaching decisive conclusions on the socio-cultural factors influencing PMTCT efforts in Nigeria. However, our use of a theoretical model, the PEN-3 cultural model enabled us to ascertain the cultural factors influencing PMTCT service uptake, including what factors are beneficial and should be encouraged and what factors harmful and should be discouraged. Challenges associated with using the PEN-3 cultural model are also well documented [80] and they include transferability, that is the extent to which the findings from our synthesis of the literature can be transferred from one context to another.

Notwithstanding these limitations, this paper contributes to an emerging body of research demonstrating the importance of socio-cultural factors in influencing PMTCT service uptake in Nigeria. To our knowledge, this paper also represents the first use of a well-known cultural model to assess how these sociocultural factors influence PMTCT in Nigeria. If scaling up access to effective PMTCT services and virtual elimination of new HIV infection among children is to be achieved by 2015, then socio-cultural factors that influence HIV testing, HIV disclosure, infant feeding choices or adherence to ARV’s must be recognized as important with reducing pediatric HIV infections in Nigeria. Furthermore, the pervasive burden of MTCT in Nigeria presents a serious public health problem requiring multiple strategies to virtually eliminate HIV among children by 2015. One such strategy is to use widely available, socially acceptable infrastructure (religious institutions) in addition to healthcare facilities as they provide access to pregnant women close to their home environment. While improvements in the accelerated provision of comprehensive PMTCT services would in all likelihood make substantial contributions to reducing the transmission of HIV through MTCT, culture-centered, congregation-based PMTCT interventions focused on the range of the perceptions people have, the enabling factors that facilitate or hinder service uptake and the role of family and/or community contexts can also reduce MTCT.

## Conclusions

Our review of the literature provide evidence to suggest that appropriate implementation of sound family-oriented, culture-centered interventions could go a long way towards achieving the comprehensive four pronged strategy currently in place in Nigeria, including: the primary prevention of HIV infection among women of reproductive age, the prevention of unintended pregnancies among HIV positive women, the prevention of HIV transmission from HIV infected mothers to their infants, and the provision of care and support for HIV infected mothers, infants and family members. Overall, the findings of the literature reviewed are congruent with the notion that culture matters with determining the level of health of the individual, family, and community context. Our findings also lend support to the idea that culture-centered interventions focused on the factors that nurture the individual including family contexts is crucial to the success of PMTCT service uptake such as HIV testing, infant feeding choices, and adherence to ARV’s. Finally, while we examine the role of culture in this paper and are also making the case for faith-based intervention research, very little is known about the intersection of culture and faith particularly in the context of families. We believe this is an important area of future research.

## Abbreviations

HBI: Healthy Beginning Initiative
PEN-3 cultural model: **P**erson, **E**xtended Family, **N**eighborhood (Cultural Identity domain): **P**erceptions, **E**nablers, and **N**urturers (Relationship and Expectation domain): **P**ositive, **E**xistential and **N**egative (Cultural Empowerment domain)
PMTCT: Prevention of mother-to-child transmission of HIV.
